# Electrical Impedance Spectroscopy as Electrical Biopsy for Monitoring Radiation Sequelae of Intestine in Rats

**DOI:** 10.1155/2013/974614

**Published:** 2013-09-04

**Authors:** Pei-Ju Chao, Eng-Yen Huang, Kuo-Sheng Cheng, Yu-Jie Huang

**Affiliations:** ^1^Department of Radiation Oncology, Kaohsiung Chang Gung Memorial Hospital, Chang Gung University College of Medicine, No. 123, Ta-Pei Road, Niao-Sung, Kaohsiung 833, Taiwan; ^2^Biomedical Imaging and Instrumentation Laboratory, Department of Biomedical Engineering, National Cheng Kung University, No. 1, University Road, Tainan 701, Taiwan; ^3^Medical Device Innovation Center, National Cheng Kung University, No. 1, University Road, Tainan 701, Taiwan

## Abstract

Electrical impedance is one of the most frequently used parameters for characterizing material properties. The resistive and capacitive characteristics of tissue may be revealed by electrical impedance spectroscopy (EIS) as electrical biopsy. This technique could be used to monitor the sequelae after irradiation. In this study, rat intestinal tissues after irradiation were assessed by EIS system based on commercially available integrated circuits. The EIS results were fitted to a resistor-capacitor circuit model to determine the electrical properties of the tissue. The variations in the electrical characteristics of the tissue were compared to radiation injury score (RIS) by morphological and histological findings. The electrical properties, based on receiver operation curve (ROC) analysis, strongly reflected the histological changes with excellent diagnosis performance. The results of this study suggest that electrical biopsy reflects histological changes after irradiation. This approach may significantly augment the evaluation of tissue after irradiation. It could provide rapid results for decision making in monitoring radiation sequelae prospectively.

## 1. Introduction

Electrical impedance is one of the most often used parameters for characterizing material properties, and it is good for use in tissue characterization. The electrical impedance of a tissue is a function associated with biological structure, including cell size, density, spacing, and the constituents of the extracellular and intracellular matrices. Besides, electrical impedance also varies with changes of applied current frequency, as employed in electrical impedance spectroscopy (EIS), revealing both the resistive and capacitive components of tissue. Schwan described the electrical properties of tissues and cell suspensions in the 1950s and concluded that electrical impedance analysis of biological media is a powerful research tool for biological research [1]. Past research has shown that variations in electrical impedance among tissues can be a good marker of pathological changes in human and animal subjects [2–11]. EIS has great potential for monitoring the tissue's change, that introduced the concept of “electrical biopsy”, and this “electrical biopsy” approach may be used to complement histological examinations [12].

Radiation therapy is a definitive treatment for malignant diseases. It uses ionizing radiation for treatment. Ionizing radiation works by damaging the DNA of exposed tissue leading to cellular death [13]. The radiation affects not only the malignant cell but also normal tissue. Therefore, evaluation of the damage of normal tissue during and after radiation therapy is very important to enhance the therapeutic benefit. The pathological damage of irradiated tissue depends on tissue properties, radiation dose, and the latent period. Radiation damage mostly occurs in cells that are actively dividing. As such, the tissues of the digestive tract, especially the mucosa, are early responding tissues that injure quickly after irradiation and are the major target of clinical radiation complications [13]. In the acute phase of radiation damage in intestine tissue, the mucosa thickens and presents with ulcerations, followed by epithelial atypia. In the latent period, vascular sclerosis and intestine wall fibrosis occur [14]. Electrical biopsy by EIS should be an idea (method) to monitor the radiation sequelae of the tissue with its' electrical characteristics.

The purpose of this study was to measure the electrical properties of intestinal tissues by electrical biopsy after whole abdomen irradiation in rats and compare them to the histology findings, for monitoring the radiation sequelae.

## 2. Materials and Methods

### 2.1. Electrical Impedance Spectroscopy System

**Figure 1:**
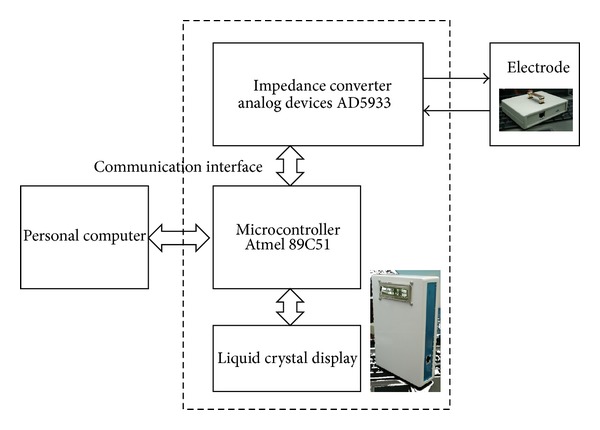
Block diagram and images of the electrical impedance spectroscopy (EIS) system. The components of the EIS system are shown. The electrode is connected to the device, and the device is controlled by a personal computer.

The EIS system for the electrical biopsy of tissues was based on an electrical impedance converter chip (AD5933) acquired from Analog Devices (Norwood, MA, USA) and a microcontroller unit (MCU; 89C51) available from Atmel Corporation (San Jose, CA, USA). The MCU received the user's instruction and relayed the measurement results to a personal computer through the universal asynchronous receiver/transmitter communication interface, and the single AD5933 chip was responsible for measuring the electrical impedance. A block diagram of the device is shown in Figure 1. The detailed system design and performance evaluation are delineated in Chen, 2007 [15].

### 2.2. Electrodes

**Figure 2:**
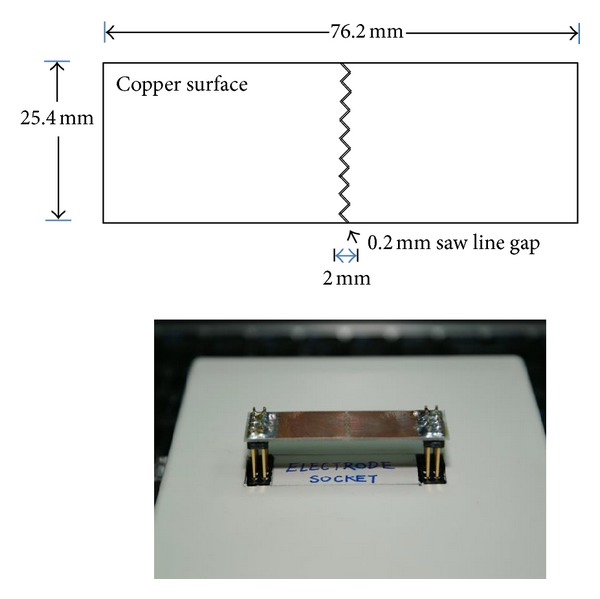
The electrodes for intestinal mucosa measurements.

The electrodes are fabricated on a printed circuit board with two parallel plates of copper, and the size is the same with a glass slide. The gap between the electrode plates is 0.2 mm with a saw line to increase the effective measurement area. A sketch and photograph of the electrodes are demonstrated in Figure 2.

### 2.3. Animal Care and Use

Specific pathogen-free Sprague-Dawley rats (male, weighing 300–350 g) were used in this study. The rats were bred in the laboratory animal center of our institution within a specific pathogen-free environment. The rats were fed with standard forage and water and caged in pairs. The rats were anaesthetized by intra-abdomen injection of thiopental (50 mg/kg) before irradiation and EIS measurements. All procedures and measurements were performed in strict accordance with protocols approved by the Animal Care and Use Committee.

### 2.4. Irradiation

The rats were immobilized in the supine position on a linear accelerator couch (Varian 2100C) after anaesthetization. The prescribed radiation dose to the whole abdomen was delivered, and the rats were kept warm using a heating light until recovery. The rats in the control group were anaesthetized simultaneously with the experimental group but not irradiated.

### 2.5. Electrical Impedance Spectroscopy Measurements

The room temperature of the laboratory was set at 22°C. The electrodes were polished using a melamine sponge and then cleaned with an alcohol swab three times before the measurements. The rats were anesthetized by intraperitoneal injection of thiopental (50 mg/kg), and the peritoneal cavity was opened by laparotomy. The distal jejunum, traced from the cecum, was dissected. A 5 cm length of distal jejunum was sampled. Subsequently, the jejunum was dissected using scissors to expand a 5 × 5 mm sample. The specimen was place on absorbent paper first to eliminate extra fluid and then pasted on the electrodes with the mucosal surface down. A cover glass was used to cover the specimen with a weight load of 20 g. The electrical impedance was measured from 10 kHz to 100 kHz at 1 kHz steps. The sample was then prepared for histological examination.

### 2.6. Histological Examination

The specimens underwent histological examination with Masson's trichrome stain. The radiation injury score (RIS), modified from Langberg et al., was used to determine the changes in histopathology [14]. There were five items assessed for morphological evaluation regarding early and late response (Table 1).

### 2.7. Experiment Design

Each check point comprised eight rats. Four were for control and four were for experiment group. The experimental groups were prescribed whole abdomen irradiation for 18 Gy, and the control groups were treated the same as the experimental group except that the linear accelerator was not turned on. The rats were sacrificed at the 8th hour, 3rd day, 9th day, 14th day, 21st day, 28th day, 35th day, and 40th day after irradiation for check. These 8 check points were annotated as 8Hrs, D3, D9, D14, D21, D28, D35, and D40, respectively. Totally, there were 64 rats for examinations. The intestinal tissue of the rats was assessed by electrical impedance measurements and histological examination after being sacrificed.

### 2.8. Data Analysis and Statistics

**Figure 3:**
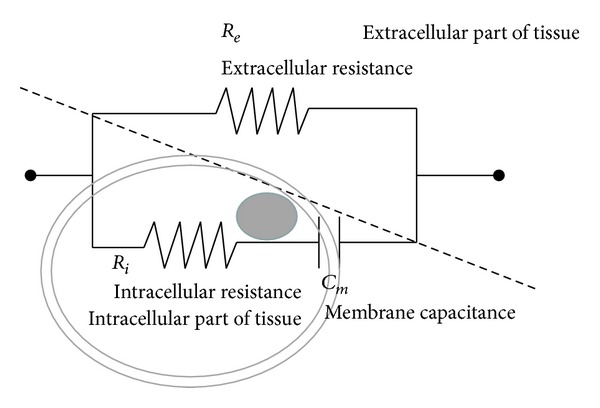
Simplified equivalent three-element resistor and capacitor (RC) electrical circuit model for tissues.

The simplified equivalent three-element resistor and capacitor (RC) electrical circuit model (as depicted in Figure 3) was applied for the interpretation of the EIS data. ZSimpWin Version 3.1 (Princeton Applied Research, Oak Ridge, TN, USA) was used to solve *R*_*e*_ (extracellular resistance), *R*_*i*_ (intracellular resistance), and *C*_*m*_ (membrane capacitance). The receiver operating characteristic (ROC) analysis was used to evaluate the diagnostic performance.

## 3. Results and Discussion

### 3.1. Results of Three-Element RC Electrical Circuit Model for Tissues

**Figure 4:**
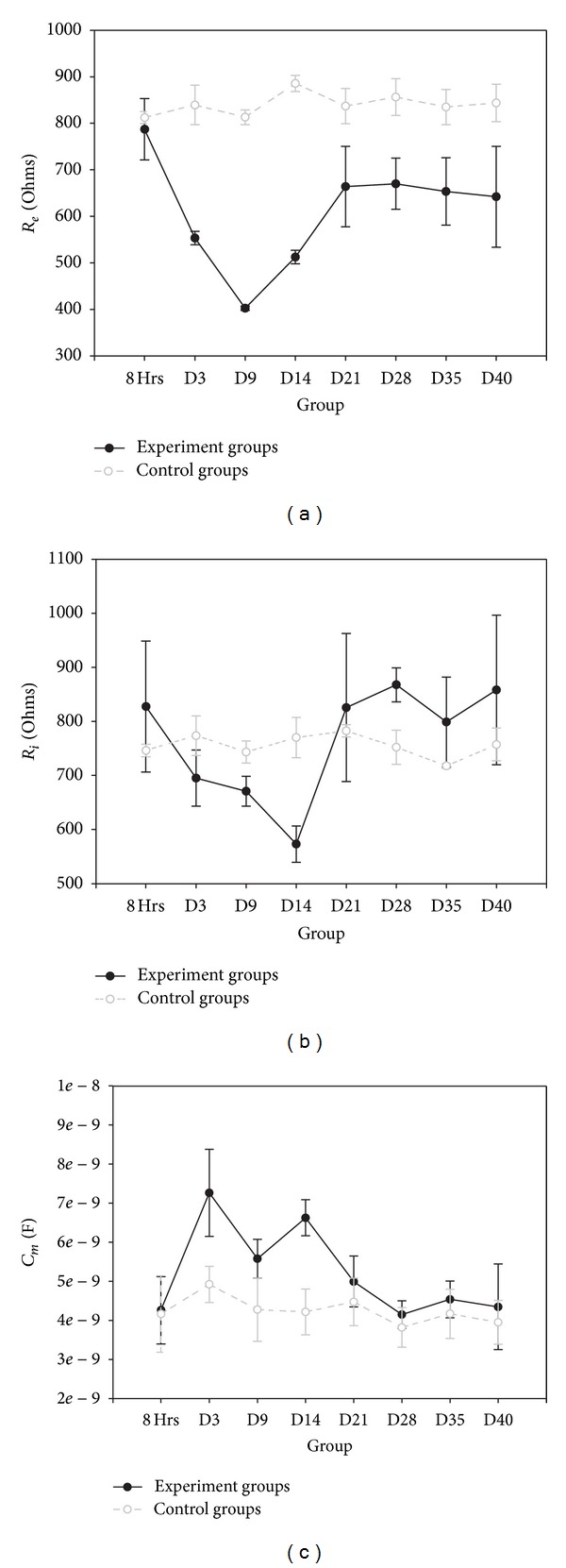
Symbol graphs of electrical parameters for electrical circuit model of tissue after irradiation. Symbol graphs showing error bars for each control and experimental group; (a) extracellular resistance (*R*_*e*_), (b) intracellular resistance (*R*_*i*_), and (c) membrane capacitance (*C*_*m*_).

The solutions to the three-element RC electrical circuit model for each group are illustrated in Figures 4(a)–4(c). *R*_*e*_ was reduced after irradiation until the 9th day. *R*_*i*_ was decreased until the 14th day and then recovered. *C*_*m*_ was increased from 3rd to 21st days. The errors of solutions established by ZSimpWin were in the range of 0.92–2.08% for *R*_*e*_, 1.39–2.23% for *R*_*i*_, and 3.30–5.24% for *C*_*m*_.

An equivalent circuit model fit derived from impedance spectroscopy can provide detailed biological information of the tissue's electrical characteristics. The most well-known model of tissue electrical characteristics is the Cole-Cole equation [16], although the parameters of this equation are not very intuitive. The three-element model, as depicted in Figure 3, is simple and easily understood from a biological viewpoint. However, the specific architecture and complex electrolytic plasma of biological tissue imply that its equivalent circuit should in principle be very complicated. The three-element model effectively eliminates any other features of the tissue and is unaware of various dispersive events. As such, all information may not be sufficiently captured by impedance spectroscopy. However, an adequate equivalent circuit model for representing tissue morphology has not been developed to date. The three-element model not only is the simplest but is often used in related studies. Although the three-element model used in this study may not be the most appropriate representation of irradiated tissues, it characterized some simple electrical characteristics of the tissues.

### 3.2. Results of Histological Examination

**Figure 5:**
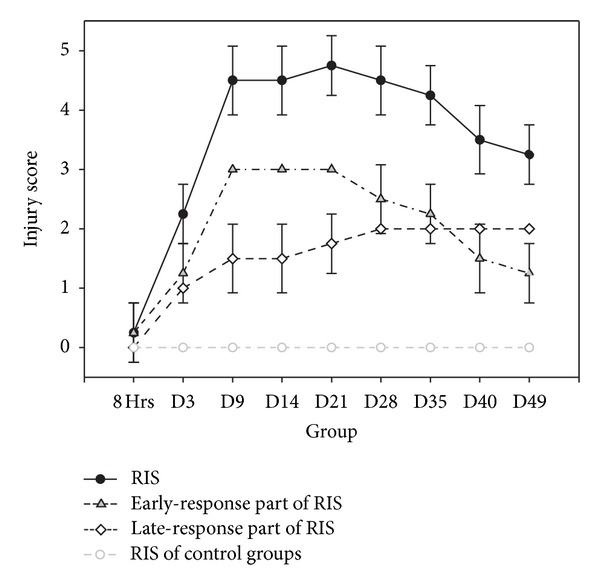
Symbol graph of radiation injury scores after irradiation. Symbol graph showing error bars of total, early-response, and late-response part of RIS after irradiation.

The results of histological examination of the control and experiment groups with RIS are shown in Figure 5. The overall RIS accumulated rapidly on the 9th day and gradually decreased after the 28th day. The early-response part of RIS subsided after the 21st day, but late-response part of RIS steadily progressed.

The vulnerability of the gastrointestinal epithelium to ionizing radiation has been well documented. Radiation enteropathy can be categorized into two stages, early and late [17]. Early radiation enteropathy occurs in the actively proliferating intestinal crypt cell compartment of intestinal mucosa, which is the primary target site of radiation injury. Stem cell depletion leads to insufficient replacement of epithelial cells in the upper crypt and villus, causing denudation of the mucosa. Following radiation exposure, the villus epithelium gradually regenerates. Late radiation enteropathy, which manifests as vascular and connective tissue damage, occurs after a variable latency period. These phenomena frequently occur in the intestinal wall instead of the mucosa. The progression of radiation injury induces morphological changes in the mucosa and the intestinal wall during the early and late stages of enteropathy, respectively. In histological examinations, as shown in Figure 5, the total postirradiation RIS score decreased after the 28th day. The acute response prevailed between the 3rd and 21st day, while the late response steadily progressed after irradiation and persisted after 28th day, are consistent.

Comparing the results of electrical parameters and the results of histological examination of tissues, the radiation sequelae of intestine in rats could be suggested to be monitored as follows.

### 3.3. Extracellular Resistance for Monitoring the Start of Radiation Sequelae

**Figure 6:**
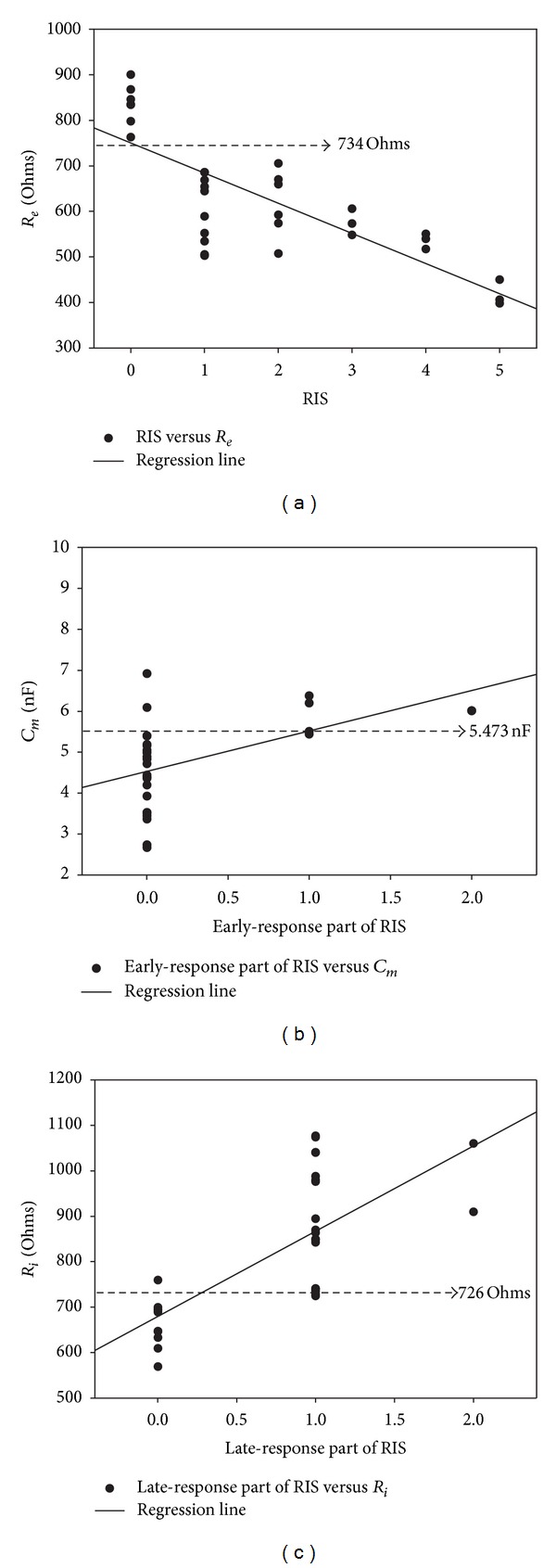
The scatter plots for electrical parameters versus radiation injury score. (a) The scatter plots for *R*_*e*_ versus RIS. *R*_*e*_ < 734 *Ω* could be used to be the threshold of the beginning of radiation injury. (b) The scatter plots for *C*_*m*_ versus early-response part of RIS. *C*_*m*_ > 5.473∗10^−9^ F could be used to be the threshold of the domination of early-response radiation injury. (c) The scatter plots for *R*_*i*_ versus late-response part of RIS. *R*_*i*_ > 726 *Ω* could be used to be the threshold of the domination of late-response radiation injury.

The scatter plot for the *R*_*e*_ versus RIS is shown in Figure 6(a). According to ROC analysis, the area under curve (AUC) for *R*_*e*_ to marke the beginning of radiation injury (RIS ≥ 1) is 1.0. *R*_*e*_ < 734 *Ω* could be used to check the beginning of radiation injury, the sensitivity and specificity are 1.0 and 1.0, respectively. *R*_*e*_ quantifies the extracellular resistance, which reflects the status of the extracellular fluid. The first morphological change after irradiation is serosa thickening, most likely a symptom of edema. The extracellular fluid accumulation leads to decreased *R*_*e*_.

### 3.4. Membrane Capacitance for Monitoring the Dominant Early-Response Radiation Injury

The scatter plot for the *C*_*m*_ versus the early-response part of RIS is shown in Figure 6(b). The AUC for *C*_*m*_ to detect the dominant searly-response radiation injury (early response part of RIS ≥ 2) is 0.95. *C*_*m*_ > 5.473∗10^−9^ F could be used to teste the dominant early-response radiation injury, the sensitivity and specificity are 0.89 and 0.93, respectively. The dominant early response presents damages such as ulceration and cell atypia. *C*_*m*_ is increased by dysfunction of the ion channels in the cell membrane due to disturbed physiological function in this stage.

### 3.5. Intracellular Resistance for Monitoring the Dominant Late-Response Radiation Injury

The scatter plot for the *R*_*i*_ versus the late-response part of RIS is shown in Figure 6(c). *R*_*i*_ indicates cytoplasm resistance. Postirradiation increases in *R*_*i*_ could be used to check the dominant late responses, which include vascular and intestinal wall fibrosis. The cytoplasm is condensed, and fibrosis leads to decreased intra- and extracellular fluids, enhancing *R*_*i*_. The AUC of *R*_*i*_ to reflect the dominant late-response injury (the late-response part of RIS ≥ 2) is 0.96. *R*_*i*_ > 726 *Ω* is the threshold to examine the dominant late-response radiation injury, the sensitivity and specificity are 0.92 and 0.90, respectively.

### 3.6. Prospect of Electrical Biopsy to Monitor Radiation Sequelae

EIS as electrical biopsy has a great potential to monitor the tissue status. The electrical biopsy by EIS indeed reflected changes of the tissue corresponding to conventional morphological findings in a sense of conventional histological knowledge [18]. In radiation therapy, the treatment responses compete with adverse effects, and monitoring the radiation sequelae and modifying treatment planning adaptively could improve the therapeutic benefit. Osterman et al. evaluated whether EIS can noninvasively determine and quantify injury responses in soft tissue exposed to high-dose rate (HDR) irradiation [19]. Small volumes of muscle tissue were irradiated with single-dose radiation using the HDR after-loading technique (26 and 52 Gy prescribed 5 mm from the source). Impedance measurements were performed on 29 rats at 1, 2, and 3 months after irradiation at 31 frequencies in the 1 kHz to 1 MHz range. Throughout the first 3 months, the conductivity increased by 48% and 26% with a target dose of 52 and 26 Gy, respectively. EIS accurately detected responses in a fraction of the tissue probed, indicating its potential usefulness in detecting radiation damage at early postirradiation time points. In human subjects for evaluation of skin tissues after irradiation, researchers have concluded that electrical impedance measurements for dielectric constants are useful for assessing cellular changes and for providing quantitative information concerning radiation-induced skin reactions [20, 21]. At 5 weeks, the dielectric constant had decreased by 31 and 39% for the investigated skin sites of the photon and electron fields, respectively. There was a statistically significant inverse correlation between the mean dielectric constant and the clinical score of erythema. Two years later, a statistically significant positive correlation was found between the dielectric constant at the irradiated skin sites and the clinical score of subcutaneous fibrosis. These support the ability of EIS as electrical biopsy to monitor the tissue status after radiation.

In future prospect, the device for electrical biopsy in monitoring radiation sequelae should be robust and easy operating. The design should be specific for clinical examination, especially of the electrodes. The electrical biopsy also has potential and capability for in situ examination. This will be helpful in real-time monitoring and treatment decision making. The electrical circuit model for individual tissue should be established, and the changes of the electrical characteristics of tissue for pathological response should be modelled more detailedly. Moreover, electrical impedance tomography (EIT) technique could evaluate the electrical parameters of tissue in body cavity without invasive procedure instantly [22]. The progression of electrical impedance technique in biomedical field has potential to provide a noninvasive, rapid results for decision making, that is not achievable in clinical evaluation and conventional pathological examination.

## 4. Conclusions

Electrical impedance spectroscopy as electrical biopsy presents as a useful method for detecting tissue injury because it reveals the electrical characteristics of tissues associated with histological change. The electrical properties were shown to accurately detect histological changes; our results demonstrated a strong diagnosis performance for radiation injury of intestine in rats. This method could be used to monitor the treatment sequelae in radiation therapy with confidence.

## Figures and Tables

**Table 1:** Morphological of assessment for evaluation of radiation injury score (RIS).

*Early response *	
Thickening of serosa	
0: None	
1: Thickening of serosa; hyperplasia of peritoneal mesothelium	
Mucosal ulcerations	
0: Small	
1: Superficial ulcerations; ulcerations involving more than half of the intestinal circumference	
Epithelial atypia	
0: None	
1: Abnormally oriented crypts; irregular crypt regeneration with atypical epithelial cells	

*Later esponse *	
Vascular sclerosis	
0: None	
1: Thickening and hyalinization of vessel wall; vessel wall double normal thickness; hyalinization and stenosis; sclerosis with stenosis or complete occlusion; fibrinoid necrosis	
Intestinal wall fibrosis	
0: None	
1: Submucosa two to four times normal thickness; broadened and hyalinized collagen fibers; abnormal collagen fibers; massive fibrosis including muscularis	
